# Prescriptions and Formulæ

**Published:** 1877-01-20

**Authors:** 


### PRESCRIPTIONS AND FORMULÆ

*Syrupus Matico et Cort. Granati.*—According to Perret (*L'Union Pharm.*,) this syrup is one of the surest and most effective astringents against dysentery, cholera morbus, acute diarrhœa, etc. It is employed either pure or diluted with water.

| | | |
|---|---|---|
| Fol. Matico........ | partes | 20 |
| Cort. graniti....... | " | 120 |
| Aquæ bullient..... | " | 1,200 |
| Sacchari............ | " | 2,000 |

The matico and pomegranate are infused with the boiling water, and allowed to stand, well covered, for twelve hours. The infusion is then filtered, and the sugar dissolved therein. Dose, a teaspoonful to a wineglassful.

*Velpeau's Diarrhœa Mixture* (*The Pharm.*)—

Tinct. opii,
Tinct. opii camph.,
Tinct. rhei.............aa fl. oz. j
Tinct, capsici........... fl. dr. vj
Spts. menthæ pip..... fl. dr. x
M.

*Haarlem Oil* (*The Pharm.*)—

Linseed oil...............2 pints.
Resin.........................1 pound.
Sulphur...................1 pound.
Oil of turpentine........1 pint.
Strong water of ammo..50 drops.
M.

"*Carbolate of Iodine*" (Dr. Percy Boulton's formula in *The Pharm.*)—

Tinct. iodinii comp......fl. dr. j
Acid. carbolici............min. vj
Glycerinæ...................fl. oz. j
Aquæ.........................fl oz, v.
M.

The solution soon loses its iodine color, becoming clear and colorless. It is used for inhalation.—*New Rem.*

R. Tinc. eucalyptus globulus,
Tin.c cinchonæ.........aa oz. ij M.
S.—Give two drachms three times a day in obstinate intermittants. W.

R. Sol. sulph. morphine.....oz. iij
Bromide potassium.......oz. ss M.
S.—One teaspoonful every two to four hours, in dysmenorrhœa, and other painful nervous affections of females.

R. Deodorized tinc. opii.........oz. ss
Bromide of ammonium......dr. ijss
M. S.—Dose, one teaspoonful every two to four hours. Excellent nervine and anti-spasmodic. Will agree with patients who cannot bear other forms of opium.

